# Success of Various Marketing Strategies for a New-to-the-Area Orthopedic Practice

**DOI:** 10.7759/cureus.18122

**Published:** 2021-09-20

**Authors:** Christopher L Antonacci, Ali M Omari, Rocco Bassora, Harlan B Levine, Ari Seidenstein, Gregg R Klein, Christopher Inzerillo, Frank G Alberta, Samir Sodha

**Affiliations:** 1 Orthopaedic Surgery, Rothman Orthopaedic Institute, Philadelphia, USA; 2 Orthopaedic Surgery, Rothman Orthopaedic Institute, Paramus, USA; 3 Orthopaedic Surgery, Rothman Orthopaedic Institute, Montvale, USA; 4 Orthopaedic Surgery, Summit Medical Group, Berkeley Heights, USA; 5 Orthopaedic Surgery, Hackensack University Medical Center, Hackensack, USA

**Keywords:** marketing, survey, practice, management, advertising, orthopedic surgery

## Abstract

Background

Competition for patients among orthopaedic private practices, multi-specialty groups, and hospital systems continues to persist. An effective marketing campaign is essential for a practice to succeed in this competitive environment. The purpose of this study was to investigate the cost-effectiveness and efficacy of each marketing campaign and the influence of patient demographics on efficacy.

Methods

The first 300 consecutive, new patients were prospectively surveyed on how they initially discovered and then selected the orthopaedic practice. Demographics and marketing costs were tabulated and categorized to analyze the effectiveness of each marketing strategy.

Results

A substantial portion of the marketing budget was allocated for traditional (67.0%) and online advertising (25.0%). However, only 56/300 (18.7%) patients surveyed were brought to the practice by these methods combined. In contrast, expenditure on a marketing liaison (8.0%) delivered 128 patients (42.7%) through referrals: 80 (26.7%) from physicians, 28 (9.3%) from urgent cares, 17 (5.7%) from physical therapists, and 3 (1.0%) from attorneys.

Conclusion

Marketing strategies were not proportionally beneficial during the first six months of the orthopaedic practice start-up period. During this early ramping up period, the most cost-effective marketing strategy was utilization of a liaison for direct in-person visits to various healthcare facilities.

## Introduction

As our demand-driven health care system evolves, patient selection of physicians appears to have become increasingly complex. Expanded enrollment in consumer-directed health plans has enabled patients to have greater choice over physician selection [1]. Social media and internet resources are becoming increasingly popular for patients and orthopaedic providers alike [2]. Given the considerable costs associated with opening a new orthopaedic practice, it is important for physicians and administrators to know where to spend valuable time and resources to attract new patients.

Several factors influencing patient selection of orthopaedic surgeons have been identified, including physician manner, surgical outcomes, board certification, in-network physician status, and reputation for expertise [1,3-6]. However, it is unclear if these findings are applicable to new orthopaedic practices not previously known to an area. New ambulatory practices may have absent, nascent, or lose affiliations with local hospital systems and associated referral networks. In light of projected increases in demand for orthopaedic services as the nation’s population continues to age [7] and the opportunity for growth into new geographic markets [8], the factors affecting patient decision-making when choosing a new orthopaedic surgery physician are increasingly relevant.

To date, there are no studies specifically analyzing the most cost-effective short-term marketing strategies to attract patients to a new orthopaedic practice. The purposes of this study were to (1) investigate which marketing campaign elements were specifically responsible for patients’ selection of a newly established orthopaedic surgery practice; (2) determine the influence of patients’ demographic characteristics on the effectiveness of the differing marketing strategies; and (3) evaluate the cost-effectiveness of each marketing strategy in its ability to deliver new patients to the practice. The authors hypothesized that expenditure on different marketing campaign components would be proportional to their effectiveness in delivering new patients to the practice.

## Materials and methods

Following Institutional Review Board approval from Thomas Jefferson University, the first 300 consecutive, new patients were prospectively surveyed by a practice representative at their first visit to ascertain how they initially discovered and then selected the new orthopaedic practice. The practice was comprised of five fellowship-trained orthopaedic surgeons (three in sports medicine, one in hand and wrist, and one in spine) with 9-14 years’ experience. The surgeons had previously been partners in an out-of-state multispecialty group practice located 48 miles from the new practice location. These surgeons subsequently joined a large group practice based in Philadelphia, Pennsylvania and represented the practice’s first expansion to northern New Jersey, where brand recognition was considered to be relatively weak. All surgeons were blinded to the allocations of the marketing budget throughout the course of the study.

Verbal responses were collated and subsequently categorized by the physician on paper. The patient’s age, gender, and town of residence were collected from the electronic medical record. Distance travelled was calculated with GoogleMaps (Mountain View, CA) using shortest distance by car from a patient’s town of residence to the practice. Patients that had been previously treated by one of the five surgeons were excluded from the study. Demographics and marketing costs for the first six months of practice activity were tabulated to analyze the effectiveness of the different marketing budget components. 

Sample size

The 300-patient sample size was selected as an estimate, based on previous practice data, of the number of new patients that would be seen in the first six months after opening. Justification of this sample size was carried out using a power analysis, where the power was calculated to be 0.96, exceeding the 0.80 threshold needed to reach power.

Marketing liaison job description

The marketing liaison’s responsibilities primarily included the following: visiting referring physicians’ offices in all target areas or territories; establishing new relationships in the territory with potential referral sources (i.e. YMCA’s, fitness centers, senior centers, sports facilities, etc.); setting up a series of physician continuing medical education lectures and community lectures that are open to the public; and participating in local community sponsorship events. 

Statistics

Differences between marketing budget allocations in relation to patient reason for selecting the practice, as well as correlations between patient reason and demographic characteristics, were evaluated with student’s t-test, analysis of variance (ANOVA), Mann-Whitney test, or chi square as statistically appropriate, and reported as p-values. All statistical analyses were conducted using R Studio, version 3.5.1 (R Foundation for Statistical Computing, Vienna, Austria).

## Results

All 300 queried patients responded with one primary reason for choosing the practice between October 2018 and March 2019. There were 155 (51.7%) females and 145 (48.3%) males. Physician referral and in-network insurance comprised 50.0% of patients’ reasons for choosing the practice. Collated responses are shown in Table 1.

**Table 1:** Determinants of patient choice for an orthopaedic practice new to the area.

Reason for Choosing Practice	Patients, n (%)
Physician referral	80 (26.7%)
In-network insurance	70 (23.3%)
Traditional advertising	29 (9.7%)
Urgent care referral	28 (9.3%)
Social media / internet search	27 (9.0%)
Word of mouth	27 (9.0%)
Emergency room referral	19 (6.3%)
Physical therapist referral	17 (5.7%)
Attorney referral	3 (1.0%)

**Figure 1:**
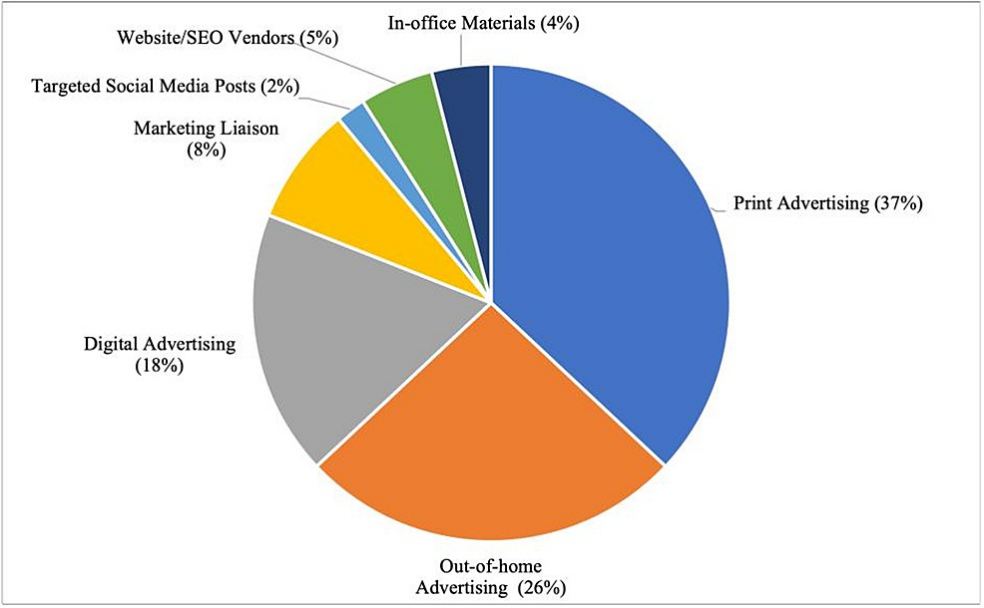
Pie chart demonstrating components of marketing budget for the first six months of practice activity. SEO: search engine optimization.

The marketing budget was comprised of seven categories: in-office materials (e.g., flyers, banners, and posters), website and search engine optimization (SEO) vendors, targeted social media posts, marketing liaison, digital advertising, out-of-home advertising (e.g. billboards, bus stops, train stations, train cars, bus wraps, malls), and print advertising. Percentage breakdown of the marketing budget in the first six months of practice activity is shown in Figure 1. Traditional advertising (print advertising, out-of-home advertising, and in-office materials) constituted 67.0% of the budget. Online advertising (digital advertising, targeted social media posts, and website/SEO vendors) comprised 25.0% of the budget. The remaining 8.0% was allocated to the marketing liaison.

**Figure 2:**
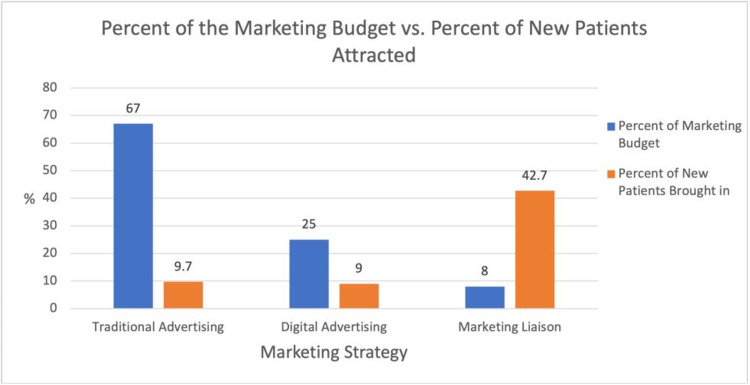
Percent of the marketing budget vs. percent of new patients attracted.

Despite a substantial portion of the budget expended on traditional and online marketing (92.0%) only 18.7% (9.7% from traditional advertising and 9.0% from social media/internet search) of the new patient cohort reported these strategies as their primary reason for practice selection. In contrast, expenditure on a marketing liaison (8.0%) was far more cost effective, delivering a total of 42.7% of the new patient cohort or 128 patients (Figure 2). These face-to-face interactions due to direct in-person marketing to the various health care establishments resulted in 26.7% (80/300) of the new patients from direct physician referral, 9.3% (28/300) more from urgent care referrals, another 5.7% (17/300) from physical therapist referrals, and 1.0% (3/300) from attorney referrals. Another 69 patients (23.1%) cited that the practice being in-network, accepting their insurance plan, as the primary driver of their selection. Word of mouth, or patients referring other patients, accounted for 9.0% and emergency room referrals represented 6.4%.

There were statistically significant differences between the number of females versus males that were referred via traditional advertising (9 vs. 20, P=0.004) and physical therapy (13 vs. 4, P=0.002) (Table 2). Patient age was significantly different between emergency room referrals and in-network patients (average age of 63.1 vs. 43.9 years, respectively; P = 0.003), emergency room referrals and word of mouth referrals (63.1 vs. 39.1 years, respectively; P = 0.001), in-network patients and physician referrals (43.9 vs. 51.5 years, respectively; P = 0.035), and physician referrals to word-of-mouth referrals (54.1 vs. 39.1 years, respectively; P = 0.012).

**Table 2:** Reason for choosing an orthopaedic practice by sex, age, and distance. Referral from an urgent care facility (UC), hospital emergency room (ER), local physician (PR), practice being in-network with patient’s insurance (IN), social media or internet review (SM), traditional advertising or print media (TA), physical therapist (PT), word of mouth (W), and an attorney (AT). *P values for comparison of referral source by gender

	AT N=3	ER N=19	IN N=69	PR N=80	PT N=17	SM N=27	TA N=29	UC N=28	W N=27	p-value
Sex:										<0.001
Female	2 (66.7%)	12 (63.2%)	33 (47.8%)	37 (46.2%)	13 (76.5%)	15 (55.6%)	9 (31.0%)	17 (60.7%)	16 (59.3%)	
Male	1 (33.3%)	7 (36.8%)	36 (52.2%)	43 (53.8%)	4 (23.5%)	12 (44.4%)	20 (69.0%)	11 (39.3%)	11 (40.7%)	
Comparison^*^	0.414	0.105	0.610	0.343	0.002	0.414	0.004	0.109	0.174	
Age	47.7 (12.1)	63.1 (23.9)	43.9 (17.5)	54.1 (17.9)	51.5 (19.5)	49.5 (12.5)	49.8 (17.2)	45.5 (24.0)	39.1 (18.5)	<0.001
Distance (miles)	50.4 (26.7)	9.14 (10.5)	10.2 (8.30)	10.3 (7.93)	6.99 (5.71)	11.7 (11.7)	11.0 (10.2)	10.6 (9.25)	17.0 (20.4)	<0.001

## Discussion

Fundamental to the success of any medical practice is a strong patient base. In order to establish this base, a novel orthopaedic practice must quickly attract new patients, while also ensuring a steady flow in the long-term. With patient selection of physicians becoming increasingly complex and the ever-expanding scope of marketing modalities available, it is important for physicians and administrators to know where to spend valuable time and resources. This study demonstrates, contrary to our hypothesis, that employment of a marketing liaison is the most cost-effective short-term marketing strategy to attract patients to a novel orthopaedic practice.

The marketing liaison was tasked to engage, via face-to-face interaction, with local physician offices, urgent care centers, and physical therapy clinics in order to increase the practice’s exposure and visibility in the market. Hiring this liaison constituted 8.0% of the marketing budget, significantly less than the amount allocated to traditional (67.0%) and online advertising (25.0%). The marketing liaison brought in 42.7% (128/300) of new patients. In contrast, traditional advertising brought in 9.7% (29/300) of new patients, while online advertising attracted 9.0% (27/300). These results demonstrate that developing relationships with referring physicians and other healthcare providers can help maximize the number of patients choosing a new orthopaedic practice previously unknown to the area.

Social media and internet searches were a relatively low influencer, with only 27 (9.0%) patients citing this as their primary method of choosing an orthopaedic provider. This appears to contrast with the relationship between social media and business commerce. A Nielsen study commissioned by Twitter demonstrated that one in four new vehicle purchases in the United States used Twitter as an input to their purchase decision; one in three of these buyers acknowledged that the platform facilitated them in making their final decision [9]. A study by Mohney et al. also found that 50% of naïve respondents, a person with no affiliation to a health-care system and no history of interaction with an orthopaedic surgeon, would use the internet to choose a physician [10]. Respondents stated that physicians that self-promote are seen as more competent, more excellent, more likely to provide quality care, and patients were more likely to choose him/her for surgery. Though we expected a younger demographic to utilize social media and internet searches, another Nielsen study demonstrated that Generation X (ages 35-49) spends more time on social media than any other group, with an average of seven hours per week [9]. The average age of our patients in the social media category (49.5 ± 12.5) is in line with these findings.

Manning et al. surveyed 231 orthopaedic spine patients to evaluate the factors that patients consider when selecting a spine surgeon and found board certification and in-network provider status to be the two most important pre-visit factors in choosing a spine surgeon; radio, internet, and television advertisements were rated least important [5]. Our study confirmed that being in network is a significant driver of patient choice. Likewise, another survey of 382 patients by Manning et al. suggested that in-network provider status, board certification, and surgeon reputation for expertise were the most important factors patients used in selecting an orthopaedic sports medicine practice [1]. Our study found that in-network provider status was the second most important factor influencing patient choice (69 patients, 23.1%), suggesting that practices new to a particular area can rely on in-network status as a significant draw for patients. The results of this study also suggest that a relatively younger population is more apt to keep in line with insurance plan participants. Startup practices should therefore consider making every effort to enter into commercial insurance plans covering the area’s patient population. 

Unlike the previously mentioned studies, our study found that physician referral was the most important factor influencing patient choice, with 80 (26.8%) patients identifying this as their primary reason for choosing their physician; this increased to 144 (48.2%) when all medical professional referrals were combined (referrals from urgent care, emergency departments, physical therapists, and specific physicians). This is an interesting finding given that referral patterns between primary care physicians and specialists often rely on previously existing personal and professional relationships [11]. It appears that at least in the early development of a practice, time still should be dedicated to cultivating relationships with local primary care physicians as well as visiting surrounding urgent care centers and physical therapist offices.

Moreover, this reliance upon physician referrals as our data suggests, is not surprising when viewed in the context of expanded enrollment of consumer-directed health plans [12]. It has been suggested that consumer-directed health plans should moderate the traditionally strong physician influence on patients’ choices of providers because referring physicians are often unaware of the cost differences to the user of referral recommendations [13]. We would expect physician referrals to be a relatively small source of new patients if a practice is out of network because consumer-directed models attempt to provide users with information to help them determine the best provider at the best price. Our finding suggests that a substantial proportion of patients continue to value their primary care physician’s advice when the provider referred participates in the patients’ health insurance company.

Limitations

This study has several limitations. First, the patient was asked by a provider during the initial encounter for the primary reason for choosing the practice. The provider’s presence may have influenced their response. Another limitation is that only the primary reason for choosing the practice was recorded, when there may have been more than one reason. We also attributed all physician referrals to the efforts of the marketing liaison as each of the physicians were on a list of providers that were directly visited. However, it is conceivable that certain physician referrals were outside this scope due to timing differences. For instance, a patient referral may have come prior to any visitation by the marketing liaison to that practice and may therefore overstate the importance of the liaison. Furthermore, the scope of this study did not include tabulating the physician referrals, so it is difficult to stratify referring physicians according to reason for referral (e.g., strategic alliance, reputation of the orthopaedic group in other geographic regions, personal relationships, etc.). This study’s reliance upon patients’ memories may also have introduced recall bias.

We analyzed only the short-term effectiveness of these marketing strategies in attracting new patients in the first six months of our practice. Patients that are referred from other healthcare providers might need more timely care and as a result, inflate the early numbers seen. Finally, traditional and social media modes of advertising promote a practice’s reputation and brand and may have a delayed impact on mid to long-term attraction of new patients. A follow up study is needed to assess how these marketing strategies change in importance over time.

## Conclusions

Our study demonstrates that traditional and online marketing are less effective than a marketing liaison in drawing patients to a start-up orthopaedic practice. Direct in-person visits to various primary care practices, urgent care facilities, and physical therapy centers resulted in the greatest proportion of new patients into the practice. Physicians and administrators in new practices should consider allocating proportional funds to the most cost-effective marketing methods in the short-term.

## References

[1] Manning BT, Bohl DD, Saltzman BM (2017). Factors influencing patient selection of an orthopaedic sports medicine physician. Orthop J Sports Med.

[2] Saleh J, Robinson BS, Kugler NW, Illingworth KD, Patel P, Saleh KJ (2012). Effect of social media in health care and orthopedic surgery. Orthopedics.

[3] Tu HT, Lauer JR (2008). Word of Mouth and Physician Referrals Still Drive Health Care Provider Choice. Res Brief.

[4] Bozic KJ, Kaufman D, Chan VC, Caminiti S, Lewis C (2013). Factors that influence provider selection for elective total joint arthroplasty. Clin Orthop Relat Res.

[5] Manning BT, Ahn J, Bohl DD, Mayo BC, Louie PK, Singh K (2016). Spine surgeon selection criteria: factors influencing patient choice. Spine.

[6] Manning BT, Bohl DD, Wang KC, Hamid KS, Holmes GB, Lee S (2018). Factors influencing patient selection of a foot and ankle surgeon. Foot Ankle Spec.

[7] Kurtz S, Ong K, Lau E, Mowat F, Halpern M (2007). Projections of primary and revision hip and knee arthroplasty in the United States from 2005 to 2030. J Bone Joint Surg Am.

[8] Weichel D (2012). Orthopedic surgery in rural American hospitals: a survey of rural hospital administrators. J Rural Health.

[9] Casey S (2021). 2016 Nielsen social media report. https://www.nielsen.com/us/en/insights/report/2017/2016-nielsen-social-media-report/.

[10] Mohney S, Lee DJ, Elfar JC (2016). The effect of orthopedic advertising and self-promotion on a naïve population. Am J Orthop (Belle Mead NJ).

[11] Kinchen KS, Cooper LA, Levine D, Wang NY, Powe NR (2004). Referral of patients to specialists: factors affecting choice of specialist by primary care physicians. Ann Fam Med.

[12] Bonney R (2005). Consumer-directed Healthcare and Its Implications for Providers.

[13] Haviland AM, Eisenberg MD, Mehrotra A, Huckfeldt PJ, Sood N (2016). Do "Consumer-Directed" health plans bend the cost curve over time?. J Health Econ.

